# Editorial: Exercise intervention for prevention, management of and rehabilitation from chronic obstructive pulmonary disease (COPD)

**DOI:** 10.3389/fphys.2023.1228431

**Published:** 2023-06-06

**Authors:** Alan Hamilton, Kay Tetzlaff

**Affiliations:** ^1^ COPD Foundation, Miami, FL, United States; ^2^Department of Health Research Methods, Evidence and Impact (HEI), Faculty of Health Sciences, McMaster University, Hamilton, ON, Canada; ^3^Department of Sports Medicine, University of Tübingen, Tübingen, Baden-Württemberg, Germany

**Keywords:** COPD-chronic obstructive pulmonary disease, exercise intervention approaches, endurance training, resistance training, non-invasive ventilation (NIV), complex intervention development, self-determination theory, behaviour change techniques

Following the inclusion of “physical reconditioning” in several COPD comprehensive care programs, as well as early studies exploring the benefits of endurance training in COPD (Petty et al., 1969; Make, 1986), the American Thoracic Society (ATS) included exercise conditioning as an “essential” component in their first pulmonary rehabilitation (PR) statement in 1981 (Hodgkin et al., 1981). Since then, exercise training has maintained a central position in the various updates to the ATS PR statement (1999 (Lareau et al., 1999), 2006 (Nici et al., 2006), 2013 (Spruit et al., 2013)). In the 1990s, the physiological mechanisms explaining the benefits of endurance training in patients with COPD began to emerge, with evidence of reduced ventilatory requirements during exercise secondary to morphologic and biochemical adaptations in skeletal muscle (Casaburi et al., 1991; Maltais et al., 1996; Maltais et al., 1997). At the same time, the recognition that many COPD patients have peripheral muscle weakness (Hamilton et al., 1995; Gosselink et al., 1996; Bernard et al., 1998) led to exploration of resistance training as an alternative form of exercise training (Simpson et al., 1992; Bernard et al., 1999; Spruit et al., 2002). Consistent with key physiological principles, adjunct therapies within exercise-based interventions have been explored to optimize the stimulus for physiological adaptations during endurance training (bronchodilators, oxygen, helium, and non-invasive ventilation) and resistance training (anabolic hormonal supplementation) (Spruit et al., 2013).

Studies included in the present Research Topic have focused on endurance training, resistance training and adjunct therapies. In exploring clinical phenotyping of exercise limitation, Gelinas et al. observed differential physiological responses to incremental exercise and proposed that patient-specific exercise limitation phenotypes may assist practitioners in prescribing a more appropriate exercise program to target the ventilatory and/or cardiovascular exercise limitation and optimize physiological adaptations. To support exercise prescription and progression in resistance training studies, Calatayud et al. evaluated the neuromuscular responses to progressive elastic band resistance in patients with COPD. With a focus on the potential respiratory/locomotor muscle interplay with adjunct therapy, Labeix et al. explored the effects of pressure ventilatory support during exercise on peripheral skeletal muscle endurance before and after an endurance training program.

***Future directions in the development and evaluation of exercise-based interventions: a perspective from the editors.*** The multidisciplinary nature of exercise-based intervention development and evaluation is highlighted by Zhou et al. who conducted a bibliometric analysis as a resource for investigators seeking research collaborators. In recent years, the development, evaluation and implementation of exercise-based interventions has become increasingly complex: there is a growing need to increase the applicability and accessibility of exercise-based interventions such as pulmonary rehabilitation; novel forms of exercise-based interventions are being developed using newer technologies that facilitate intervention delivery via teleconferencing and apps, and incorporate wearables (e.g., for physical activity) and remote monitoring (Holland et al., 2021); there is an increasing exploration of novel forms of exercise training in COPD, including interval training, upper limb training, and transcutaneous neuromuscular electrical stimulation (Spruit et al., 2013); with improvements in breathlessness and activity limitation in daily life as the ultimate target, exercise-based interventions are including behavior change techniques to influence the ability, motivation and confidence to engage in physical activity and maintain outcomes (Bourbeau et al., 2015).

In recognition of this complexity, development and evaluation of exercise-based interventions in COPD may benefit from an integrative, interdisciplinary approach embedded within a complex intervention framework (Brighton et al., 2020; Trompette et al., 2020; Skivington et al., 2021). For example, the development of the exercise-based intervention included in the PHYSACTO (Troosters et al., 2018) study was accomplished by an interdisciplinary team comprised of experts in exercise/respiratory physiology (with knowledge of the physiological principles of exercise training) and behavioural psychology (with experience in developing behaviour change interventions), with intervention development guided by a psychophysiological model of breathlessness and activity limitation in patients with COPD.

- With exercise endurance as a proximal target, intervention development was grounded in a physiological model of progressive limitation in exercise endurance over time in COPD. With expiratory flow limitation, the respiratory response to support increased metabolic demands of muscular work results in disproportionate breathlessness; to avoid breathlessness, patients reduce the intensity and/or amount of activity performed during daily life (Jones et al., 2009; Dobbels et al., 2014); reduced activity leads to muscular de-conditioning; early onset of lactic acidosis during exercise stimulates breathing, increases breathing work, increases breathlessness, and creates a downward spiral of disability (Ramon et al., 2018).
- With physical activity as a downstream target, intervention development was also grounded in behavioural theory to develop a mechanistic model of key causal assumptions connecting the included behaviour change techniques to the psychological target and downstream behavioural outcomes (Michie and Johnston, 2012). Importantly, to support sustained behaviour change in the context of a chronic disease, intervention delivery was informed by postulates from Self-Determination Theory (SDT) (Ryan and Deci, 2017) that explain the internalization of motivational regulation towards autonomous motivation (volitional engagement in an activity important and concordant with one’s values), which has positive cognitive, affective and behavioural consequences (in contrast to controlled motivation, with pressure to engage in an activity dictated by others’ expectations, monetary incentives or a sense of guilt or obligation). In accordance with SDT, motivational communication (MC) (Dragomir et al., 2021) techniques (non-judgmental, non-confrontational, collaborative and empathic) were included in the intervention to support internalization by facilitating the satisfaction of the basic psychological needs of autonomy (feeling that one is empowered and has choice), competence (feeling that one can be effective and capable), and relatedness (feeling close to, and valued by others) (Ntoumanis et al., 2021). The MC component of the intervention empowered patients to take greater responsibility for their health and wellbeing, with healthcare professionals serving as guides in the behaviour change process.

For researchers seeking to improve the physical and psychological condition of people with COPD and promote long-term adherence to health-enhancing behaviours (Spruit et al., 2013), we encourage exploration of the value gained by grounding exercise intervention research within a complex intervention framework, and adopting an integrated, interdisciplinary approach, using well-described psychophysiological mechanistic models to guide intervention development and evaluation.
